# Overexpression of the NEK9–EG5 axis is a novel metastatic marker in pathologic stage T3 colon cancer

**DOI:** 10.1038/s41598-022-26249-0

**Published:** 2023-01-07

**Authors:** Meejeong Kim, Hui Jeong Jeong, Hyun-min Ju, Ji-young Song, Se Jin Jang, Jene Choi

**Affiliations:** 1 Department of Pathology, Seoul St. Mary’s Hospital, The Catholic University of Korea College of Medicine, Seoul, Korea; 2 HiLab Clinical Laboratories, Hanaro Medical Foundation, Seoul, Korea; 3 Department of Pathology, Asan Medical Center, University of Ulsan College of Medicine, 88 Olympic-ro 43-gil, Songpa-gu, Seoul, 05505 Korea

**Keywords:** Cancer, Gastrointestinal cancer, Metastasis, Tumour biomarkers

## Abstract

NEK9 is a key player in the NEK9–EG5 axis for microtubule polymerization, chromosome alignment, and mitosis. In present study, we investigated the altered expression of the NEK9, EG5 and acetyl-α-tubulin as well as common epithelial–mesenchymal transition (EMT) markers (E-cadherin, vimentin, claudin-1, and β-catenin) through the immunohistochemistry analysis of 138 patients with pathologic T3 (pT3) stage colon cancers, and evaluated their metastatic potential. NEK9 expression showed an association with distant metastasis (*P* = 0.032) and was an independent predictive factor for distant metastasis (HR = 3.365, *P* < 0.001) by multivariate analysis, which was more significant than either the regional nodal metastasis (HR = 2.496, *P* = 0.007) or lymphovascular invasion (HR = 2.090, *P* = 0.153). Positive correlations were observed between NEK9 and EG5 or acetyl-α-tubulin (r = 0.236 and *P* = 0.007; r = 0.181 and *P* = 0.038, respectively) and concordant overexpression of the NEK9–EG5 axis was further confirmed in colon cancer cell lines. These findings collectively suggest that the overexpression of the NEK9–EG5 axis is present and associated with distant metastasis in colon cancer. These biomarkers might be useful for predicting metastatic potential among the patients with pT3 colon cancers.

## Introduction

Colorectal cancer (CRC) is the third most prevalent cancer worldwide, comprising 8% of all adulthood malignant tumors, and is the second leading cause of cancer-related mortality (5.8% of the total deaths)^1,2^. Although the CRC mortality rate had decreased with improvements in treatment, the 5-year relative survival rate for patients with stage IV (i.e., distant metastatic colon cancers) remained lower than 15% compared with 91% for T1–T3M0 and 72% for T4M0^3^.

The NEK family is a founding member of never-in-mitosis A (NIMA) protein family and 11 Neks (NEK1–11) have now been described, many of which have cell cycle-dependent activity^4^. Of these factors, NEK9 is active in the regulation of mitotic spindle assembly and functions during mitosis along with NEK6 and NEK7, thereby constituting a mitotic kinase cascade in which NEK6 and NEK7 are phosphorylated and activated by NEK9 and contribute to mitotic progression^5^. Several studies have described the altered expression of NEK proteins in various cancers. Decreased NEK9 expression has been reported to correlate with the aggressive behavior of breast cancer, whereas NEK7 upregulation was shown to be related to a poor prognosis in both gastric and pancreatic cancer^6–8^. In CRC, NEK2 or NEK6 overexpression are frequently observed and are associated with poor outcomes^9,10^. Hence, the aberrant expression of the NEK family proteins needs to be further understood with regard to tissue-specific tumorigenesis.

EG5 (known as kinesin-5) is phosphorylated and activated by NEK6/7, which are activated by NEK9. Thus, NEK9 is part of an NEK9–EG5 axis via the recruitment of EG5 to the centrosome to control mitosis and subsequent microtubule elongation (polymerization)^11^. In terms of microtubule dynamics, the acetylation of α-tubulin at lysine 40 (K40) is the only post-translation modification that occurs in the lumen of the microtubules, and is found in stable and long-lived microtubules as it enhances their self-repair and protection from mechanical breakage^12^. Despite the known regulatory of NEK9, EG5 and microtubule acetylation in mitotic events, few studies have investigated the prognostic significance of these factors in different tumor types. A recent study by O’Regan et al. has reported that microtubule stabilization through NEK9 and NEK7 leads to increased cancer migration, and that a correlation exists between NEK9 and acetylated tubulin expression^13^.

Although little is yet known about CRC metastasis specifically, tumor metastasis is generally associated with cell proliferation, motility involving an epithelial–mesenchymal transition (EMT), mitosis, and microtubule dynamics^14–16^. Here, we investigated the altered expression of NEK9, EG5, and acetyl-α-tubulin, and their correlation with the EMT-related proteins (E-cadherin, claudin-1, vimentin, and β-catenin) to evaluate their role and possible prognostic significance for metastasis in patients with pathologic T3 stage (pT3) colon cancers.

## Results

A series of 138 patients with colon adenocarcinoma of a pT3 stage were included in the study cohort, while 51 of 138 patients developed distant metastasis (stage IV). Their clinicopathologic features are summarized in Supplementary Table 1. The mean age of these patients was 63.6 years (range, 31–90 years) and the male to female ratio was 1:1.03. Survival data were available in 136 of these cases. Median follow-up period was 46 months (range, 0–155 months) and two patients were lost to follow-up. The enrollment periods of the two patients with 0 months follow-up were 18 days in both cases. One patient lost to follow-up was excluded from the survival analysis. Thirty-eight patients died during follow-up and the mean overall survival (OS) of the study population was 113.73 months (95% CI 102.80–124.66).

### Expression of the NEK9–EG5 axis and clinicopathologic features

**Figure 1:**
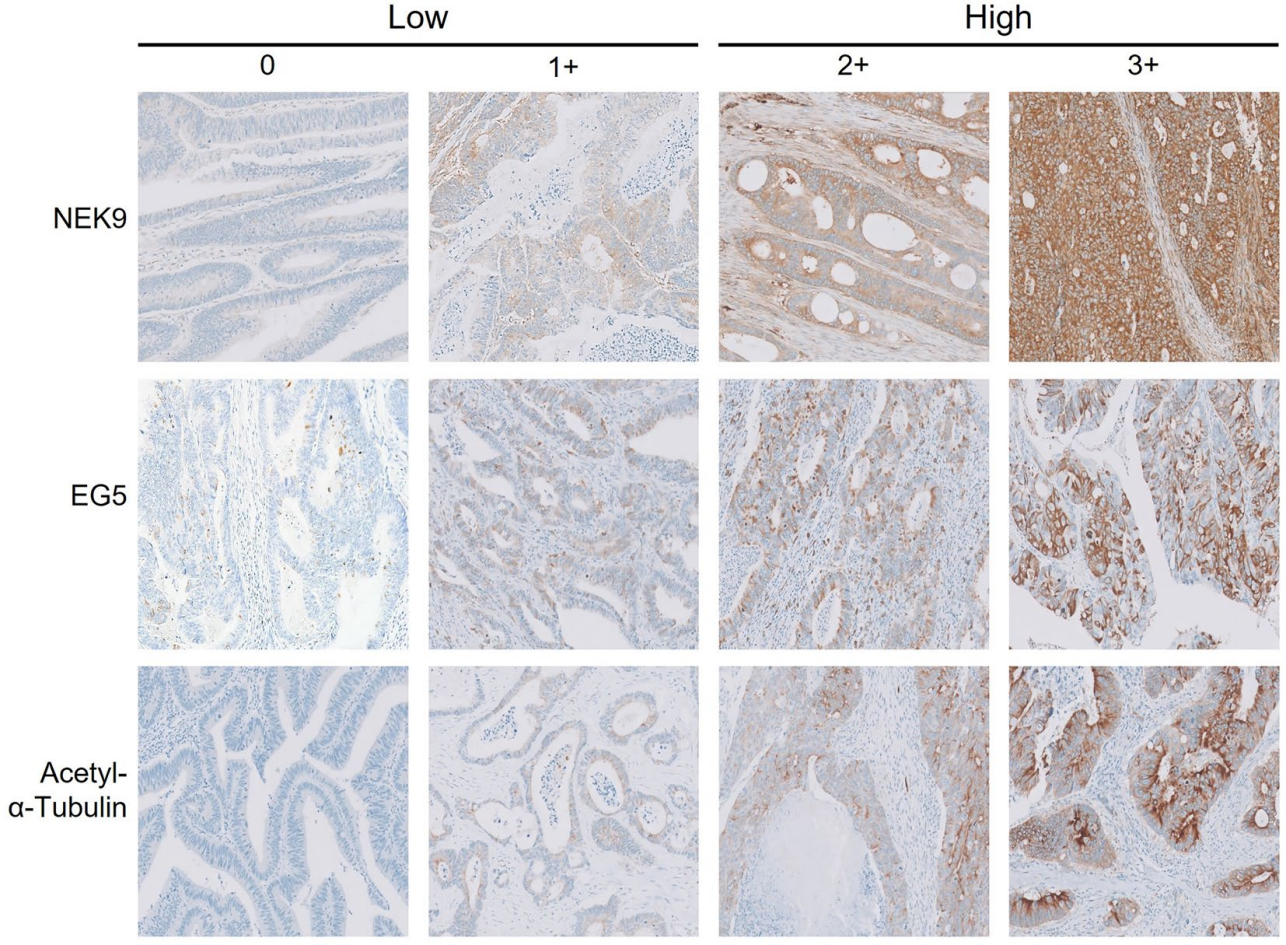
Representative images of immunostained tissue microarrays from the colon adenocarcinoma samples with immunoreactivity scores for NEK9, EG5, and acetyl-α-tubulin (magnification, × 200). NEK9, EG5, and acetyl-α-tubulin were thereby grouped into low- (score 0–1) or high- (score 2–3) expression groups.

Overall, 69% (91/132), 53% (70/132), and 74% (98/132) of our present study cases showed high expression for NEK9, EG5 and acetyl-α-tubulin by IHC analyses. Representative images and details of the IHC scores are provided in Fig. 1 and Supplementary Table 2, respectively. The CRC tumors showing high NEK9 expression were preferentially distributed among patients with M1 stage tumors (*P* = 0.032). In addition, a significant correlation was observed between high NEK9 expression and a tumor size greater than 60 mm (*P* = 0.046). High EG5 expression also showed a significant association with the LVI (*P* = 0.009). The high expression of EG5 or acetyl-α-tubulin was predominantly found in the M1 group, but these results did not reach statistical significance (*P* = 0.107 and 0.111, respectively; Table 1). Compared with NEK9, the EMT markers (E-cadherin, vimentin, claudin-1 and ꞵ-catenin) were not found to be significantly associated with the M1 stage in the present study cohort, but the expression of E-cadherin or ꞵ-catenin did show an association with tumor differentiation (*P* = 0.037 and 0.002, respectively; Supplementary Table 3). Representative images of EMT marker staining are presented in Supplementary Fig. 1.

**Table 1:** Immunohistochemical expression of NEK9, EG5 and acetyl-α-tubulin and clinicopathologic characteristics.

Variables (n, %)	NEK9		*P*	EG5		*P*	Acetyl-α-tubulin		*P*
	Low (n = 41)	High (n = 91)		Low (n = 62)	High (n = 70)		Low (n = 34)	High (n = 98)	
**Size**			0.046			0.520			0.984
≤ 60 mm	34 (82.9)	60 (65.9)		42 (67.7)	51 (72.9)		24 (70.6)	69 (70.4)	
> 60 mm	7 (17.1)	31 (34.1)		20 (32.3)	19 (27.1)		10 (29.4)	29 (29.6)	
**N stage**			0.767			0.996			0.989
N0	21 (51.2)	41 (45.1)		28 (45.2)	32 (45.7)		16 (47.1)	45 (45.9)	
N1	14 (34.1)	33 (36.3)		23 (37.1)	26 (37.1)		12 (35.3)	36 (36.7)	
N2	6 (14.6)	17 (18.7)		11 (17.7)	12 (17.1)		6 (17.6)	17 (17.3)	
**M stage**			0.032			0.107			0.111
M0 (stage III)	31 (75.6)	51 (56.0)		43 (69.4)	39 (55.7)		25 (73.5)	57 (58.2)	
M1 (stage IV)	10 (24.4)	40 (44.0)		19 (30.6)	31 (44.3)		9 (26.5)	41 (41.8)	
**Differentiation**			0.382			0.239			0.063
WD	2 (4.9)	6 (6.6)		6 (9.7)	2 (2.9)		4 (11.6)	4 (4.1)	
MD	37 (90.2)	84 (92.3)		55 (88.7)	66 (94.3)		28 (82.4)	93 (94.9)	
PD	2 (4.9)	1 (1.1)		1 (1.6)	2 (2.9)		2 (5.9)	1 (1.0)	
**LVI**			0.188			0.009			0.617
Absent	24 (58.5)	42 (46.2)		38 (61.3)	27 (38.6)		18 (52.9)	47 (48.0)	
Present	17 (41.5)	49 (53.8)		24 (38.7)	43 (61.4)		16 (47.1)	51 (52.0)	
**PNI**			0.211			0.662			0.981
Absent	20 (48.8)	55 (60.4)		36 (58.1)	38 (54.3)		19 (55.9)	55 (56.1)	
Present	21 (51.2)	36 (39.6)		26 (41.9)	32 (45.7)		15 (44.1)	43 (43.9)	
**Tumor budding**			0.116			0.527			0.391
Absent	18 (54.5)	56 (70.0)		30 (62.5)	43 (68.3)		16 (59.3)	58 (68.2)	
Present	15 (45.5)	24 (30.0)		18 (37.5)	20 (31.7)		11 (40.7)	27 (31.8)	

*WD* well differentiated, *MD* moderately differentiated, *PD* poorly differentiated, *LVI* lymphovascular invasion, *PNI* perineural invasion.

### Expression of the NEK9–EG5 axis, metastasis, and overall survival

By univariate analyses, the expression of NEK9 (hazard ratio [HR] = 2.476, 95% confidential interval [CI] 1.520–4.033, P < 0.001), claudin-1 (HR = 0.561, 95% CI 0.336–0.937, P = 0.027), the N stage (HR = 2.563; 95% CI 1.545–4.251, P < 0.001), LVI (HR = 2.971; 95% CI 1.442–6.122, P = 0.003) and the PNI (HR = 2.199; 95% CI 1.087–4.450, P = 0.028) were found to be significantly associated with distant metastasis (Table 2). Multivariate analyses revealed that NEK9 expression was the independent predictive factor for distant metastasis (HR = 3.365; 95% CI 1.786–6.340, *P* < 0.001), which was more significant than the N stage (HR = 2.496, 95% CI 1.284–4.852, *P* = 0.007), while the EMT markers (E-cadherin, vimentin, claudin-1 and ꞵ-catenin) did not show this predictive capacity with any significance (Table 2).

**Table 2:** Univariate and multivariate regression analysis of metastasis biomarkers in the study cohort.

Variables	Metastasis			
	HR	95% CI		*P*
**Univariate**				
Size ≥ 60 mm	0.841	0.391	− 1.806	0.657
N stage (N2 vs N1 vs N0)	2.563	1.545	− 4.251	< 0.001
Differentiation (PD vs MD vs WD)	0.942	0.275	− 3.226	0.924
LVI	2.971	1.442	− 6.122	0.003
PNI	2.199	1.087	− 4.450	0.028
Tumor budding	0.481	0.216	− 1.072	0.073
NEK9	2.476	1.520	− 4.033	< 0.001
EG5	1.139	0.881	− 1.472	0.320
Acetyl-α-tubulin	1.293	0.866	− 1.932	0.209
E-cadherin	0.757	0.455	− 1.261	0.285
Vimentin	0.440	0.088	− 2.210	0.319
Claudin-1	0.561	0.336	− 0.937	0.027
ꞵ-Catenin	1.885	0.191	− 18.633	0.588
**Multivariate**				
Size ≥ 60 mm	1.237	0.460	− 3.327	0.673
N stage	2.496	1.284	− 4.852	0.007
LVI	2.090	0.761	− 5.739	0.153
PNI	1.496	0.562	− 3.983	0.420
NEK9	3.365	1.786	− 6.340	< 0.001
EG5	0.917	0.648	− 1.298	0.625
Acetyl-α-tubulin	1.397	0.823	− 2.372	0.216
E-cadherin	0.537	0.266	− 1.087	0.084
Vimentin	0.335	0.05	− 1.912	0.218
Claudin-1	0.554	0.286	− 1.073	0.080
ꞵ-Catenin	2.156	0.110	− 42.298	0.613

*WD* well differentiated, *MD* moderately differentiated, *PD* poorly differentiated, *LVI* lymphovascular invasion, *PNI* perineural invasion.

**Figure 2:**
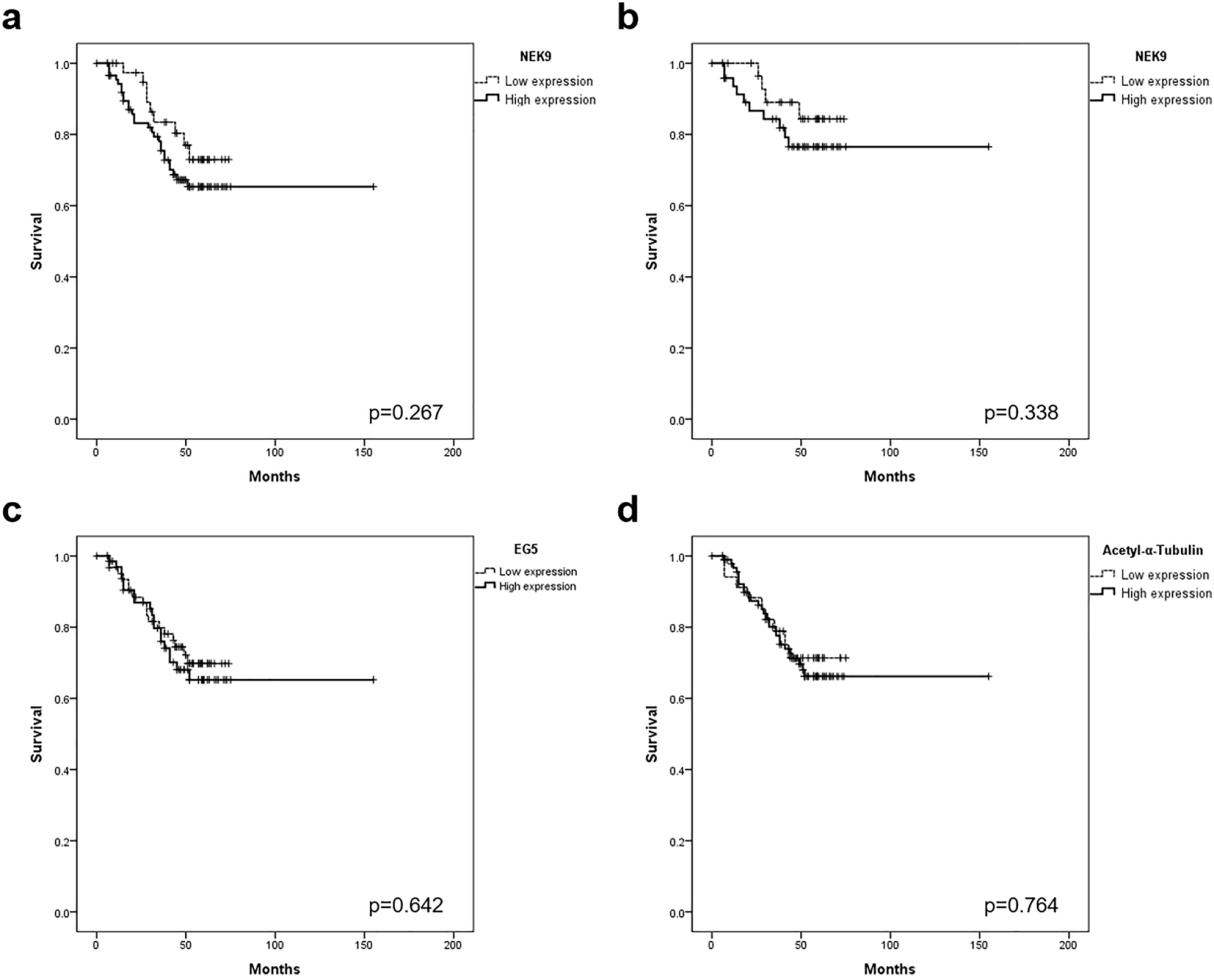
Kaplan–Meier survival curves for overall survival (OS) according to the expression of NEK9, EG5 or acetyl-α-tubulin. (**a**) NEK9 expression in the whole cohort (n = 136). (**b**) NEK9 expression in the M0 patient subgroup (stage III; n = 87). (**c**) EG5 expression in the whole cohort. (**d**) Acetyl-α-tubulin expression in the whole cohort. OS differences were observed between the low- and high-expression cases for these proteins, with a more distinct difference for NEK9. M0, patients with no evidence of metastasis.

Kaplan–Meier survival analyses demonstrated OS differences between the low and high NEK9 expression groups among our current series of 136 patients, but these were not statistically significant (*P* = 0.267; Fig. 2a). Intriguingly, these different survival outcomes were similarly observed between the two groups when limited to the M0 cases (n = 87) (*P* = 0.338; Fig. 2b). OS differences were also observed in relation to the EG5 and acetyl-α-tubulin expression levels among the total patient population (*P* = 0.642, and 0.764, respectively; Fig. 2c,d), whereas in the case of the EMT markers, only claudin-1 showed difference (*P* = 0.151; Supplementary Fig. 2). In a total of 138 patients, adjuvant fluoropyrimidine-based chemotherapy was applied in 87 (63.0%): 42 of 87 (48.3%) with stage III, and 45 of 51 (88.2%) patients with stage IV. When we performed analyses based on the adjuvant chemotherapy stages with these marker expressions, there was no statistically significant difference in OS.

### Immunohistochemical correlations and activity of the NEK9–EG5 axis in colon cancer cell lines

Using a Pearson’s test, a significant positive correlation was found between NEK9 and EG5, and between NEK9 and acetyl-α-tubulin (r = 0.236 and *P* = 0.007; r = 0.181 and *P* = 0.038, respectively). Among the M1 stage subgroup of 51 patients, NEK9 and EG5 showed a significant positive correlation (r = 0.350 and *P* = 0.013) and EG5 and acetyl-α-tubulin also did so with marginal significance (r = 0.276 and *P* = 0.053; Table 3).

**Table 3:** Correlation between NEK9, EG5, and acetyl-α-tubulin immunostaining in the study cohort.

Immunohistochemistry	Total (n = 138)		M1 (stage III; n = 51)		M0 (stage IV; n = 87)	
	r	*P*	r	*P*	r	*P*
NEK9–EG5	0.236	0.007	0.350	0.013	0.146	0.197
NEK9–Acetyl-α tubulin	0.181	0.038	0.155	0.283	0.156	0.165
EG5–Acetyl-α tubulin	0.154	0.079	0.276	0.053	0.085	0.452

*M1* patients with distant metastasis, *M0* patients with no evidence of distant metastasis.

**Figure 3:**
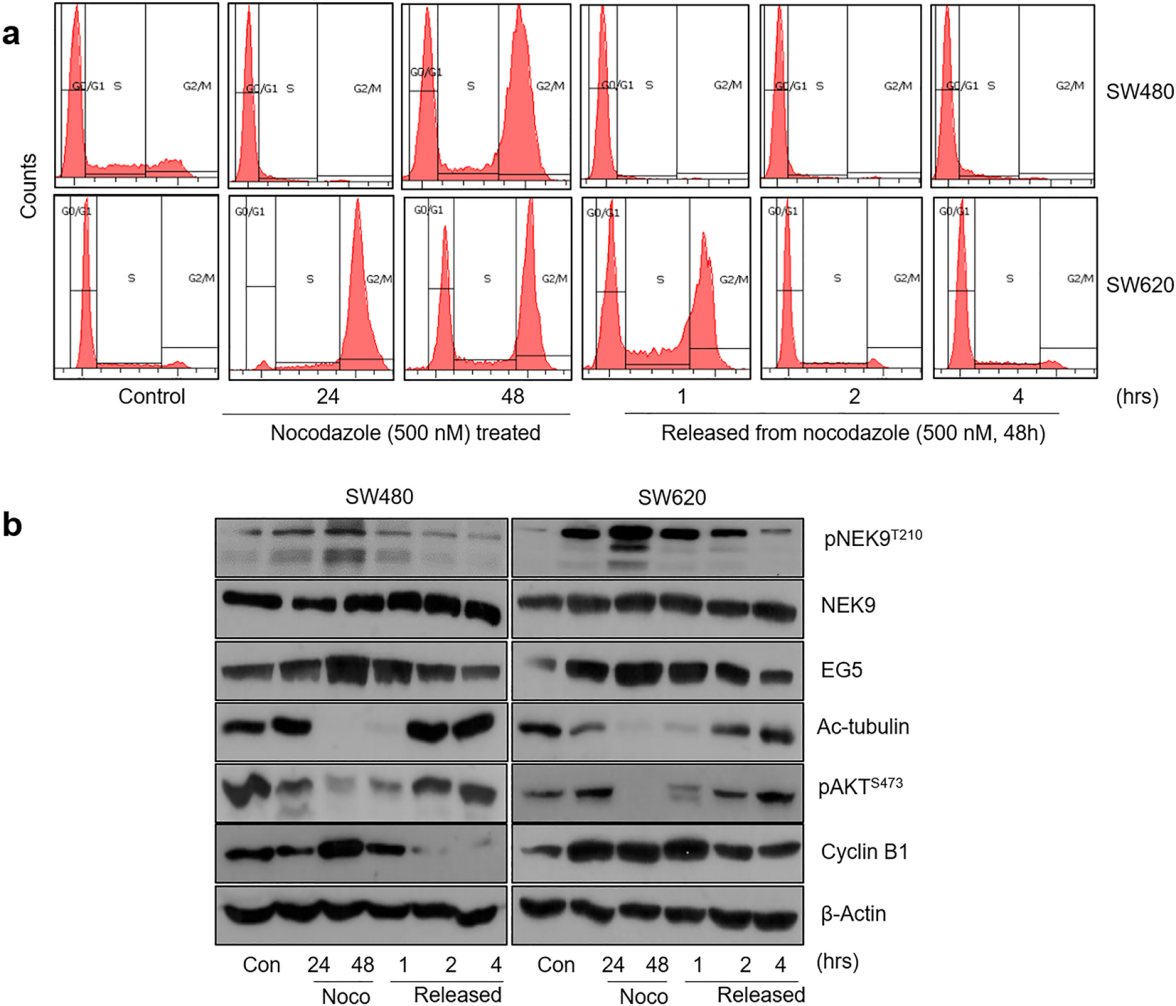
Concordant expression of the NEK9–G5 axis during the G_2_/M phase of the cell cycle. (**a**) Cell cycle distribution of SW480 and SW620 colon cancer cells treated with nocodazole (500 nM) for 24 or 48 h and then released from this block. (**b**) Western blot analysis of phosphorylated NKE9 (pNEK9^T210^), NEK9, EG5, acetyl-tubulin, phosphorylated AKT (pAKT^S473^) and cyclin B1 at the indicated times of nocodazole–induced G_2_/M arrest, and after the release from this block.

Considering that NEK9, EG5, and acetyl-α-tubulin are key cell cycle proteins controlling the G2 to M phase transition, we further confirmed their concomitant expression in cell lines. SW480 and SW620 colon cancer cells were arrested at the G_2_/M border by 48 h of nocodazole treatment and then released to enter mitosis^17,18^. SW480 cells passed transitioned from G_2_/M to G1 more rapidly than SW620 cells after this nocodazole release (Fig. 3a). The levels of cyclin B1, active NEK9 (pNEKT210) and EG5 were concordantly accumulated at the G_2_/M phase by nocodazole treatment and decreased when the cells were released into G1 (Fig. 3b). By contrast, the acetylated-α-tubulin and pAKTS473 levels were simultaneously decreased at the G_2_/M phase in which microtubule depolymerization and subsequent chromosome segregation occur. These data suggest that the NEK9–EG5 axis is active, and that the simultaneous high expression of NEK9 and EG5 is required for mitosis in colon cancer cells.

## Discussion

NEK9 expression has been recently studied in relation to carcinogenesis and tumor progression, but its oncogenic role remains controversial as evidence of both anti- and pro-tumoral effects in different types of cancers have been reported^6,19^. NEK9 is involved in microtubule polymerization, centrosome separation via the NEK6–NEK7–EG5 activation, and microtubule acetylation mechanisms that play an essential role in appropriate cell proliferation and in the segregation of the mitotic spindle for chromosome stability. To our knowledge, no prior study has investigated the NEK9–EG5 axis and microtubule acetylation as a possible metastatic marker in colorectal cancer. In this study, we have demonstrated that NEK9 could be a factor for distant metastasis in patients with pT3 colon cancer.

Cancer cells go through morphology transformation via EMT processes with associated increases in cell motility, reduced intercellular adhesion, and a loss of cell polarity, thereby acquiring metastatic characteristics. Among the related proteins involved in EMT progression, a loss of E-cadherin and overexpression of vimentin are generally indicative of invasive tumor behavior and distant metastasis^20^. In addition, recent studies have indicated that the decreased membranous expression of ꞵ-catenin involves early malignant transformation through the EMT and claudin-1, a tight junction protein, promotes an invasive phenotype^21,22^. Nevertheless, in our cohort, NEK9 overexpression was found to be an independent prognostic indicator of distant metastasis and might be a superior marker to the EMT proteins. Moreover, the poor OS tendency in the high NEK9 group was also found in the M0 patient subgroup (Fig. 2b), indicating that NEK9 overexpression may have a crucial role in the onset of distant metastasis in colon cancer.

Kurioka et al. showed that high NEK9 expression is associated with a poor prognosis in patients with non-small cell lung cancers lacking a functional p53, suggesting that this expression promotes tumor growth^23^. O’Regan et al. reported that NEK9 overexpression was associated with poor progression-free survival in patients with EML4-ALK lung cancer, and that this expression may have high metastatic potential^13^. In another recent study by Lu et al*.*, a simultaneous elevation of the levels of NEK9, GP130 and p-STAT3 in the lymph nodes was associated with distant metastasis and a reduced OS outcome in gastric cancer^24^. Taken together, these findings have suggested that the aberrant expression of NEK9 is frequently observed in various cancers and is potentially a negative prognostic indicator.

We here further investigated the relationship between NEK9 expression and well characterized clinicopathologic parameters related to a poor prognosis in colon cancer, i.e. N stage, M stage, LVI, PNI, and tumor budding and differentiation. Interestingly, our findings indicated that a high NEK9 expression level was associated with a tumor size of over 60 mm and with an M stage (Table 1). A prior large population-based study also found that a lesion size above 60 mm in colon cancer correlated with a poor prognosis^25–27^. In addition, Luo et al. demonstrated that an increased tumor size was correlated with a distant metastasis in rectal cancer^28^. Considering that NEK9 is a centrosome binding protein during mitosis, a large tumor volume could be affected by a high mitotic activity. In this regard, NEK9 might be a comparable metastatic biomarker to other pathological signs of metastatic invasion, including the LVI and PNI.

A number of studies have suggested that acetylated microtubules are stable and long-lived microtubules which are consequences of microtubule acetylation^29^. There are several reports showing concordant activation of the NEK9–EG5 axis in the mitotic phase of the cell cycle^30,31^. Our present data demonstrated that NEK9 and EG5, and NEK9 and acetyl-α-tubulin, have a positive expression correlation. Moreover, these relationships were found to be more distinct in the M1 group than among the M0 cases in our study cohort. Based on these data, we have found that an aberrant hyper-activation of the NEK9–EG5 axis, as well as stable acetylated microtubules, confer an increased metastatic potential in colon cancer. Multiple prior studies have shown that EG5 or acetyl-α-tubulin overexpression is associated with a poor prognosis^30,32,33^. Indeed, although the results did not reach statistical significance, we also observed that high NEK9, EG5, and acetyl-α-tubulin expression were associated with a poor OS outcome, with a more obvious tendency in the M0 group, i.e., stage III with high NEK expression. Hence, we conclude that the NEK9–EG5 axis could play a functional role in tumor cell proliferation and distant metastasis.

At the molecular level, concordant NEK9 and EG5 accumulation at the G_2_/M phase by nocodazole treatment were verified in colon cancer cell lines in our current experiments. However, we observed a marked decrease in acetyl-α-tubulin at the G_2_/M phase, whereas expression of the NEK9–EG5 axis showed an inverse relationship with acetyl-α-tubulin. We believe that G_2_/M synchronized cancer cells are primed for microtubule depolymerization for mitosis, and then acetylation of microtubules occurs right after mitosis to recover their structural integrity. This finding has not been described in earlier studies and the detailed mechanisms of tubulin acetylation during mitosis require further characterization^34,35^.

In this study, we showed that high NEK9 expression could be an independent metastatic marker in colon cancer, and aberrant expression of the NEK9–EG5 axis had a role in microtubule assembly with acetylation during the tumorigenic process. Mitotic phase- and microtubule acetylation-specific proteins could be included as components of the mechanisms underlying metastasis, migration and invasiveness of tumor cells.

## Materials and methods

### Patients

A cohort of 138 patients diagnosed with pT3 colon adenocarcinoma, based on the 8th edition of the American Joint Committee on Cancer (AJCC) system, between 2015 and 2017 at Asan Medical Center was enrolled in this study. Of the consecutive cases, patients who underwent a curative resection without neoadjuvant therapy were selected and any with rectal cancer were excluded. The medical records of these cases were reviewed to extract data such as sex, age, radiologic findings, survival and pathologic information. The pathologic variables that were reviewed included tumor size, histologic grade, regional lymph node metastasis, lymphovascular invasion (LVI), perineural invasion (PNI), and tumor budding. Using TNM staging system according to the 8th edition of the AJCC, N and M categories were defined as follows; N0, no regional lymph node metastasis; N1, metastasis in 1–3 regional lymph nodes or no regional lymph nodes are positive but there are tumor deposits in the subserosa, mesentery or nonperitonealized pericolic tissues; N2, metastasis in 4 or more regional lymph nodes; M0, no distant metastasis by imaging; no evidence of tumor in other sites or organs; M1, distant metastasis by radiologic imaging and pathologic tissue confirmation.

### Tissue microarrays and immunohistochemistry

Hematoxylin and eosin-stained slides of the 138 study cases were reviewed by two experienced pathologists (MK and HJJ). Tissue microarrays (TMAs) were constructed from the formalin-fixed paraffin-embedded (FFPE) tissue blocks with two randomly selected tumor cores per case measuring 3 mm in diameter. Immunohistochemical (IHC) staining was performed on these TMA blocks using a Ventana BenchMark XT Autostainer (Ventana Medical Systems, Tucson, AZ) and an UltraView staining kit (Ventana). Immunostaining of representative FFPE sections from normal colon tissue and colon adenocarcinomas were evaluated to optimize the antibody dilutions.

The samples were incubated with each of the following primary antibodies: NEK9 (1:500; ab138488, Abcam, UK), EG5 (1:100; 4203, Cell Signaling Technology, Boston, MA), acetyl-α-tubulin (1:1000; 5335, Cell Signaling Technology), E-cadherin (1:200; clone 4A2C7, Zymed, CA), claudin-1 (1:100; #359A-15, Cell Marque, CA ), vimentin (1:500, clone V9, Zymed), and ꞵ-catenin (1:200, clone 14, Cell Marque).

The results from the immunostained cores were interpreted by the two pathologists who scored the intensity (i.e. no staining, weak, moderate, and strong) and proportion of IHC staining. If two separate cores of the same case exhibited different staining results, these were averaged. Since NEK9 expression has been rarely evaluated previously and showed a diffuse expression pattern, we adopted the interpretation criteria of Lennartz and colleagues^36^. For NEK9, four scoring categories were utilized as follows: 0, no detectable staining; 1 +, weak staining intensity in ≤ 70% or a moderate intensity in ≤ 30% of the tumor cells; 2 +, weak staining intensity in > 70% or a moderate intensity in > 30% to ≤ 70%; 3 +, moderate intensity in > 70% or strong intensity in > 30% of the tumor cells. There was no case of strong intensity on NEK9 in < 30% of the tumor cells in our cohort. Although the expression pattern of acetyl-α-tubulin expression has been rarely studied, it was very similar to EG5 in our experiments; therefore, we applied the same criteria. For the scoring of EG5 and acetyl-α-tubulin, four staining categories were applied as follows: 0, no staining or staining in < 10% of the tumor cells; 1 +, faint or weak staining in ≥ 10% of the tumor cells; 2 +, weak to moderate staining in ≥ 10% of the tumor cells; 3 +, moderate to strong staining in ≥ 10% of the tumor cells^37^. Cytoplasmic or membrane IHC expression of the EMT-related proteins (E-cadherin, claudin-1, vimentin, and β-catenin) was also evaluated. For E-cadherin and claudin-1, the scores were assigned as follows: 0, no staining; 1 +, ≤ 33% positive; 2 +, > 33% to ≤ 66% positive; 3 +, > 66% positive^38^. For vimentin, detectable expression in ≥ 5% of the tumor cells was considered high and < 5% as low^39,40^. Membranous expression of β-catenin was classified as high when > 80% of the tumor cells were positively stained and otherwise as low^41^.

Those categories were used for logistic regression analysis and Pearson’s correlation tests. For cross analysis in which relationships between the IHC expression profile and clinicopathologic features were investigated, the NEK9, EG5, acetyl-α-tubulin, E-cadherin, and claudin-1 expression categories were divided into low- (score 0–1) or high-expression (score 2–3) subgroups.

### Cell culture and cell cycle analysis

Primary tumor-derived SW480 and metastasis-derived SW620 colon cancer cell lines were grown in RPMI 1640 (Invitrogen-GIBCO, Carlsbad, CA) supplemented with 10% fetal bovine serum, 50 μg penicillin/ml, and 100 μg streptomycin/ml at 37 °C in a 5% CO_2_ incubator. Nocodazole was purchased from Sigma-Aldrich (SML1665, St. Louis, MO) and stock solutions were prepared in DMSO and stored at − 20 °C. Cells were treated with either DMSO or 500 nM nocodazole for 24 or 48 h, and then released by a switch to normal media to allow re-entry into the cell cycle. For cell cycle analysis, cells were harvested and collected by centrifugation. Briefly, 5 × 10^5^ cells were washed with ice-cold PBS, fixed in 50% ethanol (vol/vol), and treated 5 μl of a 1 mg/ml RNase A. The cells were stained with 50 μl of a 50 μg/ml propidium iodide (PI) solution for 15 min on ice and then subjected to flow cytometry. DNA histograms were subsequently analyzed using BD FACSCantoTM (BD Biosciences, San Jose, CA).

### Western blot analysis

For western blotting, whole-cell lysates were prepared in RIPA lysis buffer (50 mM Tris–HCl [pH 8.0], 150 mM NaCl, 0.5 mM EDTA, 1 mM DTT, 0.1% NP-40, 0.1% SDS) containing a protease inhibitor cocktail (BPI-9200, Tech & Innovation™, Bucheon, Korea) and a phosphatase inhibitor cocktail (45065; Santa Cruz, Santa Cruz, CA). Proteins were then separated on an 8% or 10% SDS-PAGE gel and transferred to polyvinylidene fluoride (PVDF) membranes using an iBlotTM dry blotting system (Invitrogen). The transferred membranes were cut after the blocking steps to analyze with different antibodies. Immunoblotting analysis were performed with the following primary antibodies: pNEK9 (T210) (1:5000, ab63553; Abcam), NEK9 (1:5000, ab138488; Abcam), EG5 (1:1000, 4203; Cell signaling Technology), acetyl-α-tubulin (Lys40) (1:1000, T7451; Sigma-Aldrich), pAKT (S473) (1:1000, 9271; Cell signaling Technology), AKT (1:1000, 9272; Cell signaling Technology), cyclin B1 (1:1000, sc-245; Santa Cruz) and β-Actin (1:10,000, 5441; Sigma-Aldrich). Rabbit or mouse horseradish peroxidase (HRP)-labelled secondary antibodies (1:10,000; ENZO, Farmingdale, NY) were then used. The blots were visualized using a SuperSignal West Pico Chemiluminescent Substrate (Thermo Fisher Scientific-Pierce, Rockford, IL).

### Statistical analysis

Cross-tab analysis using the chi-square and Fisher’s exact tests was performed to evaluate the relationship between IHC expression and clinicopathological characteristics. OS was calculated from the date of diagnosis to death or the last follow-up visit, analyzed with the Kaplan–Meier method and compared using the log-rank test. Univariate and multivariate logistic regression analyses were performed to evaluate the relationship between the onset of distant metastasis in colon cancer and the clinicopathologic variables, including the IHC expression profiles. Pearson’s correlation coefficients were calculated to investigate the possible existence of correlations among the detected expression patterns.

All statistical analyses were performed using SPSS software package version 21.0.0 (SPSS Statistics software, IBM Corp, NY). A P-value of < 0.05 was considered statistically significant.

### Ethics statement

This retrospective study was approved by the Institutional Review Board (IRB) of Asan Medical Center with a waiver of informed consent (IRB No. 2021-1877) and was performed in accordance with the 1964 Helsinki Declaration and its later amendments.

## Supplementary Information

Supplementary Information 1.

Supplementary Information 2.

## Data Availability

Data are available from the corresponding author upon reasonable request.
