# Supplementary figures and images for: Overexpression of the NEK9–EG5 axis is a novel metastatic marker in pathologic stage T3 colon cancer
Source: Sci Rep. 2023 Jan 7;13:342. doi: 10.1038/s41598-022-26249-0 (PMC9825400; doi:10.1038/s41598-022-26249-0)

# SW480

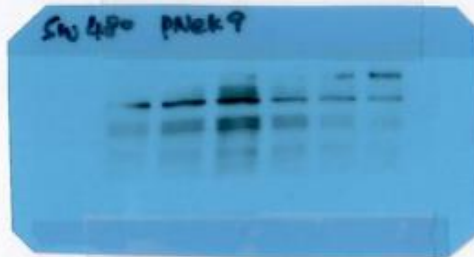

pNEK9

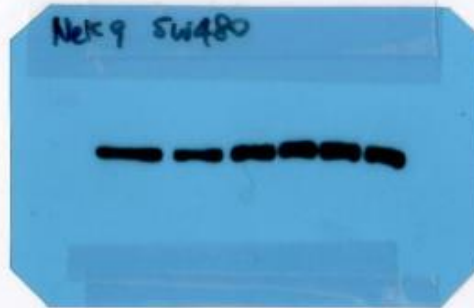

NEK9

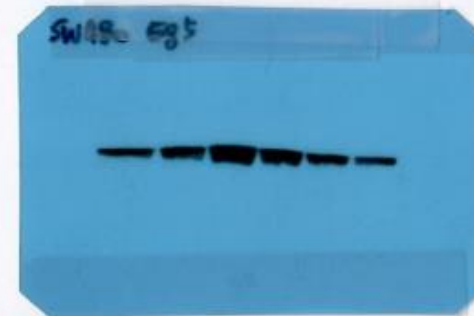

EG5

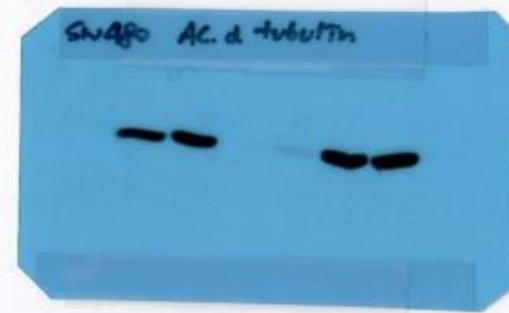

Ac.tubulin

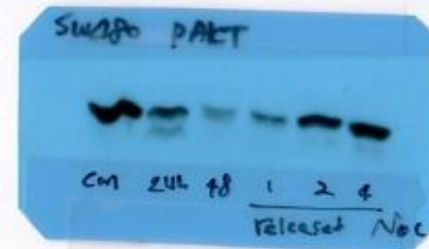

pAKT

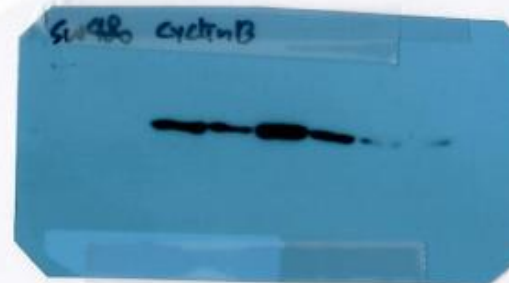

Cyclin B1

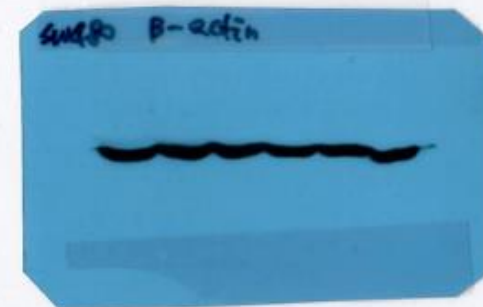

$\beta$ -Actin

# SW620

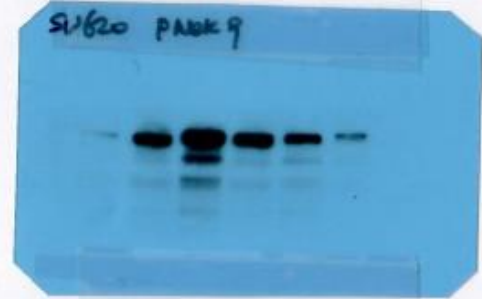

pNEK9

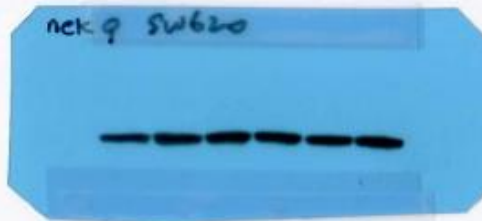

NEK9

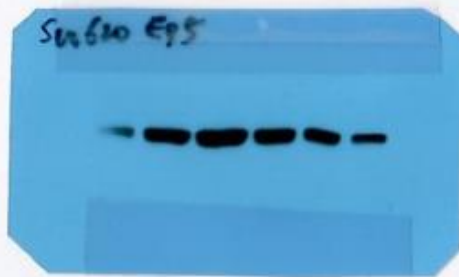

EG5

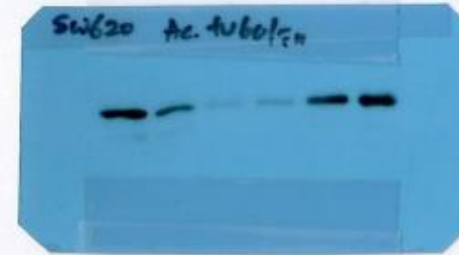

Ac.tubulin

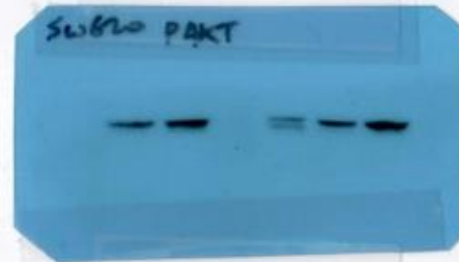

pAKT

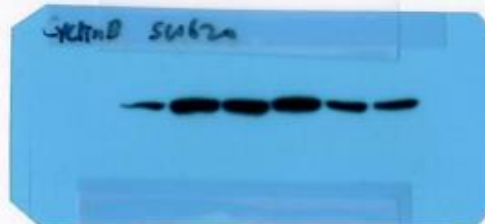

Cyclin B1

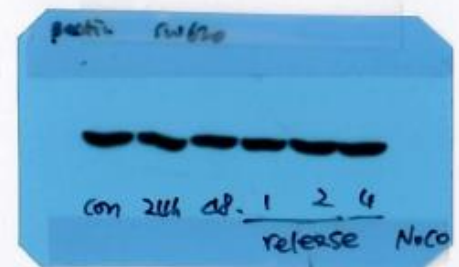

β-Actin

Supplement: Supplementary file 1 — Supplementary Information 1. [file 41598_2022_26249_MOESM1_ESM.pdf]
