# Supplementary material for: Overexpression of the NEK9–EG5 axis is a novel metastatic marker in pathologic stage T3 colon cancer
Source: Sci Rep. 2023 Jan 7;13:342. doi: 10.1038/s41598-022-26249-0 (PMC9825400; doi:10.1038/s41598-022-26249-0)
Supplement: Supplementary file 2 — Supplementary Information 2. [file 41598_2022_26249_MOESM2_ESM.pdf]

Supplementary information

Overexpression of the NEK9-EG5 axis is a novel metastatic marker in pathologic stage T3 colon cancer

Meejeong Kim[1], Hui Jeong Jeong[2], Hyun-min Ju[3], Ji-young Song[3], and Se Jin Jang[3*], and Jene Choi[3*]

[1]*Department of Pathology, Seoul St. Mary's Hospital, The Catholic University of Korea College of Medicine, Seoul, Korea.*

[2]*HiLab Clinical Laboratories, Hanaro Medical Foundation, Seoul, Korea.*

[3]*Department of Pathology, Asan Medical Center, University of Ulsan College of Medicine, Seoul, Korea.*

*Correspondence should be addressed to:

Se Jin Jang, M.D., Ph.D.

Department of Pathology, University of Ulsan College of Medicine, Asan Medical Center, 88 Olympic-ro 43-gil, Songpa-gu, Seoul 05505, Korea

Telephone: +82-2-3010-5966

E-mail: jangsejin@amc.seoul.kr

Fax: 82-2-472-7898

Jene Choi, Ph.D.

Department of Pathology, University of Ulsan College of Medicine, Asan Medical Center, 88 Olympic-ro 43-gil, Songpa-gu, Seoul 05505, Korea

Telephone: +82-2-3010-4555

E-mail: jenec@amc.seoul.kr

Fax: 82-2-472-7898

Supplementary Table 1. Baseline characteristics in the 138 patients

Characteristics	N (%)
Size	
≤ 60 mm	97 (70.3)
> 60 mm	41 (29.7)
N stage	
N0	63 (45.7)
N1	52 (37.7)
N2	23 (16.7)
M stage	
M0 (stage III)	87 (63.0)
M1 (stage IV)	51 (37.0)
Differentiation	
WD	8 (5.8)
MD	127 (92.0)
PD	3 (2.2)
LVI	
Absent	69 (50.0)
Present	69 (50.0)
PNI	
Absent	79 (57.2)
Present	59 (42.8)
Tumor budding	
Absent	76 (65.5)
Present	40 (34.5)
Adjuvant chemotherapy	
Applied	87 (63.0)
M0 (Stage III)	42 (30.4)
M1 (Stage IV)	45 (32.6)
Not applied	51 (37.0)

Supplementary Table 2. Immunohistochemical expression profiles and the frequency reactivity scores

Immunohistochemistry		N (%)
NEK9	0	0 (.0)
	1	41 (29.7)
	2	47 (34.1)
	3	44 (31.9)
EG5	0	60 (43.5)
	1	2 (1.4)
	2	21 (15.2)
	3	49 (35.5)
Acetyl-α	0	26 (18.8)
	1	8 (5.8)
	2	84 (60.9)
	3	14 (10.1)
E-cadherin	0	1 (.7)
	1	15 (10.9)
	2	61 (44.2)
	3	54 (39.1)
Vimentin	Low (< 5%)	122 (88.4)
	High (≥ 5%)	9 (6.5)
Claudin-1	0	0 (.0)
	1	15 (10.9)
	2	26 (18.8)
	3	90 (65.2)
β-catenin	Low	4 (2.9)
	High (> 80%)	127 (92.0)

Supplementary Table 3. Immunohistochemical expression of epithelial-mesenchymal transition markers and clinicopathologic characteristics

	E-cadherin			Vimentin			Claudin-1			β-catenin		
**Variables**	**Low (n=16)**	**High (n= 115)**	***P***	**Low (n=122)**	**High (n=9)**	***P***	**Low (n=15)**	**High (n=116)**	***P***	**Low (n=4)**	**High (n=127)**	***P***
**Size**			0.833			0.318			0.379			0.045
≤ 60 mm	11 (68.8)	82 (71.3)		87 (71.3)	5 (55.6)		12 (80.0)	80 (69.0)		1 (25.0)	91 (71.7)	
> 60 mm	5 (31.3)	33 (28.7)		35 (28.7)	4 (44.4)		3 (20.0)	36 (31.0)		3 (75.0)	36 (28.3)	
**N stage**			0.318			0.026			0.889			0.300
N0	10 (62.5)	52 (45.2)		58 (47.5)	2 (22.2)		6 (40.0)	54 (46.6)		3 (75.0)	57 (44.9)	
N1	3 (18.8)	43 (37.4)		41 (33.6)	7 (77.8)		6 (40.0)	42 (36.2)		0 (.0)	48 (37.8)	
N2	3 (18.8)	20 (17.4)		23 (18.9)	0 (.0)		3 (20.0)	20 (17.2)		1 (25.0)	22 (17.3)	
**M stage**			0.247			0.308			0.199			0.582
M0 (stage III)	12 (75.0)	69 (60.0)		74 (60.7)	7 (77.8)		7 (46.7)	74 (63.8)		3 (75.0)	78 (61.4)	
M1 (stage IV)	4 (25.0)	46 (40.0)		48 (39.3)	2 (22.2)		8 (53.3)	42 (36.2)		1 (25.0)	49 (38.6)	
**Differentiation**			0.037			0.144			0.818			0.002
WD	3 (18.8)	5 (4.3)		8 (6.6)	0 (.0)		1 (6.7)	7 (6.0)		1 (25.0)	7 (5.5)	
MD	12 (75.0)	108 (93.9)		112 (91.8)	8 (88.9)		14 (93.3)	106 (91.4)		2 (50.0)	118 (92.9)	
PD	1 (6.3)	2 (1.7)		2 (1.6)	1 (11.1)		0 (.0)	3 (2.6)		1 (25.0)	2 (1.6)	
**LVI**			0.616			0.784			0.712			0.332
Absent	9 (56.3)	57 (49.6)		60 (49.2)	4 (44.4)		8 (53.3)	56 (48.3)		1 (25.0)	63 (49.6)	
Present	7 (43.8)	58 (50.4)		62 (50.8)	5 (55.6)		7 (46.7)	60 (51.7)		3 (75.0)	64 (50.4)	
**PNI**			0.605			0.493			0.193			0.431
Absent	10 (62.5)	64 (55.7)		67 (54.9)	6 (66.7)		6 (40.0)	67 (57.8)		3 (75.0)	70 (55.1)	
Present	6 (37.5)	51 (44.3)		55 (45.1)	3 (33.3)		9 (60.0)	49 (42.2)		1 (25.0)	57 (44.9)	
**Tumor budding**			0.581			0.187			0.779			0.692
Absent	7 (58.3)	67 (66.3)		70 (67.3)	3 (42.9)		9 (69.2)	64 (65.3)		3 (75.0)	70 (65.4)	
Present	5 (41.7)	34 (33.7)		34 (32.7)	4 (57.1)		4 (30.8)	34 (34.7)		1 (25.0)	37 (34.6)	

WD, well differentiated; MD, moderately differentiated; PD, poorly differentiated; LVI, lymphovascular invasion; PNI, perineural invasion

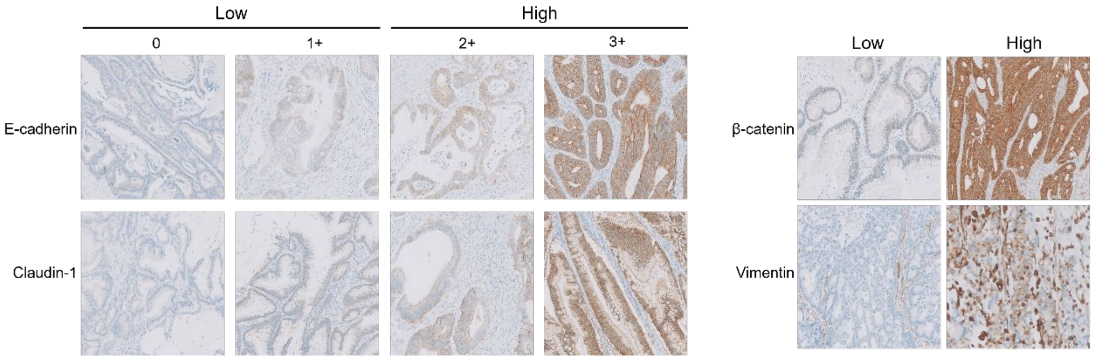

Supplementary Figure 1 Representative images of stained tissue microarrays of colon adenocarcinomas used to determine the immunoreactivity scores for E-cadherin (magnification, x200), claudin-1 (x200), β-catenin (x200), and vimentin (x200 in low- and x400 in high-expression). These scores for E-cadherin and claudin-1 were divided into the low- (score 0 – 1) or high-expression (score 2 – 3) groups.

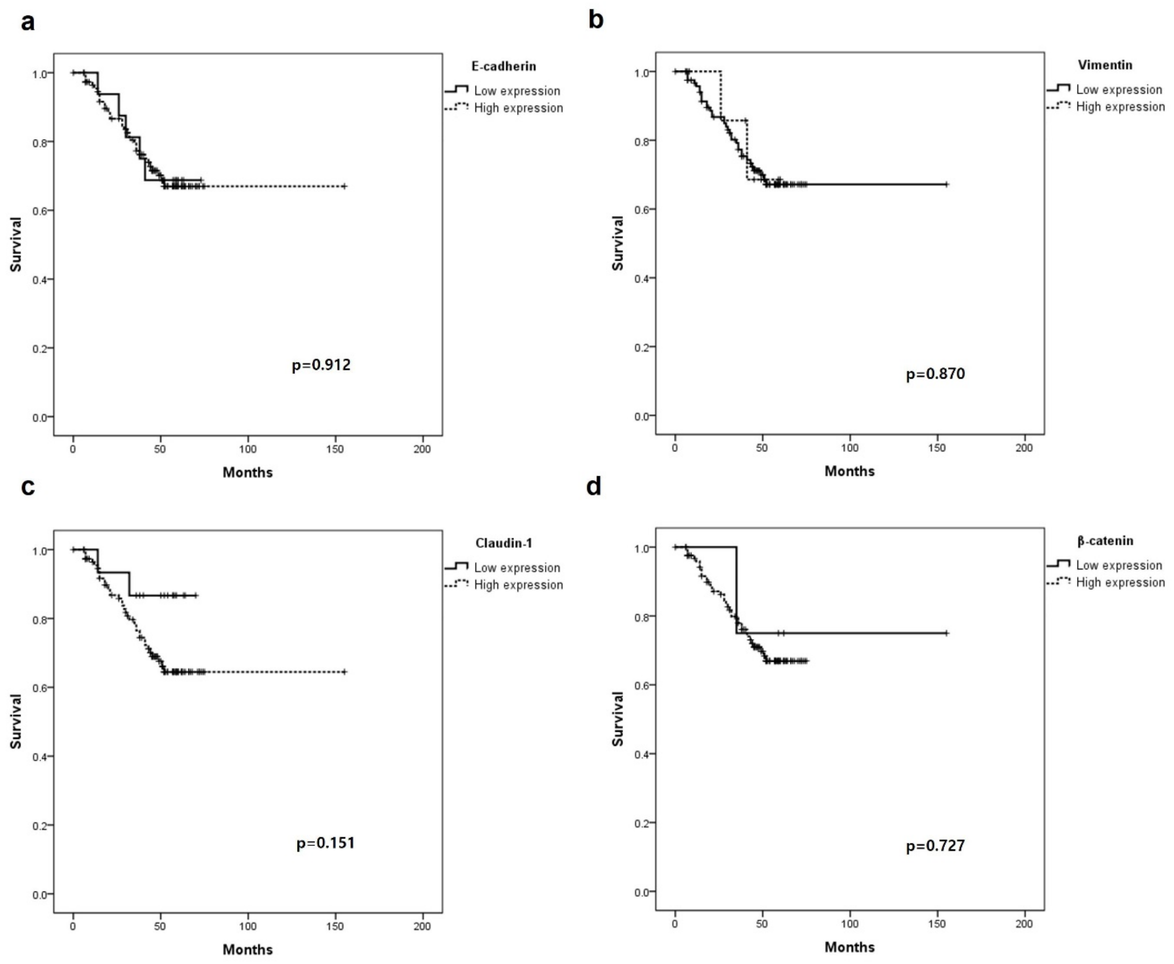

Supplementary Figure 2 Kaplan-Meier survival curves based on the immunohistochemical staining of (a) E-cadherin, (b) vimentin, (c) claudin-1, and (d) β-catenin.
